# Mechanistic Insights Into the Interaction Between Transcription Factors and Epigenetic Modifications and the Contribution to the Development of Obesity

**DOI:** 10.3389/fendo.2018.00370

**Published:** 2018-07-06

**Authors:** Qi Huang, Chaoyang Ma, Li Chen, Dan Luo, Rui Chen, Fengxia Liang

**Affiliations:** ^1^Department of Acupuncture and Moxibustion, Hubei University of Chinese Medicine, Wuhan, China; ^2^Department of Rehabilitation, The Central Hospital of Wuhan, Tongji Medical College of Huazhong Science and Technology University, Wuhan, China; ^3^Department of Traditional Chinese Medicine, Huazhong University of Science and Technology Tongji Medical College, Wuhan, China; ^4^Department of Integrated TCM and Western Medicine, Union Hospital, Tongji Medical College of Huazhong Science and Technology University, Wuhan, China

**Keywords:** obesity, transcription factors, epigenetics, DNA methylation, histone modifications, chromatin remodeling, non-coding RNA, interaction

## Abstract

**Objective:** The development of obesity is inseparable from genetic and epigenetic factors, and transcription factors (TFs) play an essential role in these two mechanisms. This review analyzes the interaction of TFs with epigenetic modifications and the epigenetic mechanisms underlying peroxisome proliferator-activated receptor (PPAR)γ, an important transcription factor, in the development of obesity.

**Methods:** We describe the relationship between TFs and different epigenetic modifications and illustrate the several mechanisms described. Next, we summarize the epigenetic mechanisms of PPARs, an important class of transcription factors involved in obesity, that induce obesity with different triggering factors. Finally, we discuss the mechanisms of epigenetic modification of PPAR-related ligands in lipid metabolism and propose future avenues of research.

**Results:** TFs participate in epigenetic modifications in different forms, causing changes in gene expression. The interactions between the different epigenetic modifications and PPARs are important biological developments that affect fat tissue differentiation, lipogenesis, and lipid metabolism, thereby inducing or inhibiting the development of obesity. We then highlight the need for more research to understand the role of epigenetic modifications and PPARs.

**Conclusions:** Epigenetic mechanisms involved in the regulation of PPARs may be excellent therapeutic targets for obesity treatment. However, there is a need for a deeper understanding of how PPARs and other obesity-related transcription factors interact with epigenetic modifications.

## Introduction

Obesity is currently the world's most conspicuous nutritional disease. It is currently considered a serious threat to human health because of its high prevalence (1). The expansion of fat mass, adipocyte size increase, and to a lesser extent cell proliferation (hyperplasia) are important features of obesity (2). The increase in adipose tissue corresponds to increase in size and number of adipocytes (3), i.e., lipogenesis and differentiation. Factors that regulate fat tissue differentiation such as insulin, growth hormone, and glucocorticoids promote differentiation of pre-adipocytes (4, 5) and genetic and transcriptional regulation (6, 7). Among regulatory factors, transcription factors (TFs) play a critical role in the regulation of adipocyte differentiation. Studies have shown that lipogenesis and differentiation are regulated coordinately by several TFs, mainly including the peroxisome proliferator-activated receptor γ (PPARγ), CCAAT enhancer binding proteins (C/EBPs), and the transcription factor sterol regulatory element binding protein-1 (SREBP-1) (8, 9). PPARγ is the most critical TF for the regulation of adipocyte differentiation, being a necessary and sufficient condition for the differentiation of adipose tissue (10). The CAAT/enhancer family has the function of activating CAAT repeats of specific gene DNA enhancers. Its family members, C/EBPα, C/EBPβ, and C/EBPδ, are considered important factors in adipocyte differentiation. C/EBPδ and C/EBPα can induce the expression of the PPARγ gene (11), which is probably achieved through a transcriptional effect of the C/EBP binding site in the PPARγ promoter (12). Activated PPARγ, in turn, increases the expression of the C/EBPα gene. In the absence of C/EBPα, cells can only express PPARγ at low levels and cannot form adipocytes (13). In mammals, SREBP is classified into two types: SREBP1 and SREBP2. SREBP1 is also known as adipocyte determination and differentiation-dependent factor 1 (ADDl), and can act independently as a TF to regulate adipocyte differentiation and transcription of genes related to cholesterol metabolism (14). Studies have shown that SREBP promotes the synthesis of triglycerides by up-regulating the expression of genes such as fatty acid synthase and lipoprotein lipase (14). SREBP can also activate PPAR expression or induce the expression of an endogenous ligand of PPAR to increase its lipogenic activity and promote adipocyte differentiation (15). In addition to the above TFs, cyclic AMP response element binding protein (CREB), nuclear factor of activated T cells (NFAT), kruppel-like factor (KLF) family, and forkhead transcription factor (FOX) proteins have been demonstrated to affect adipogenesis (16, 17).

However, the complex mechanisms of adipocyte differentiation remain to be further elucidated (18). Epigenetics is a likely candidate to elucidate the missing links that lead to obesity. Specific dietary behavior leads to change in epigenetic patterns and regulates gene expression via epigenetic modifications (19, 20). These changes interfere with metabolic homeostasis and result in body adiposity (21, 22). Epigenetic changes in chromatin also occur during adipocyte differentiation (23, 24). Some studies indicated that the epigenetic changes at the gene locus of a specific TF correlate with the body-mass index (BMI) in humans (25, 26). This suggests that the development of obesity may be inseparable from the interaction between TFs and epigenetic modification. However, it is still mostly unclear how cells establish the connection between TFs and epigenetic modifications and how it leads to regulation of gene expression. Several specific mechanisms in biological processes, from epigenetic changes to gene regulation, are still not fully understood. A systematic investigation of the link between TFs and epigenetics will provide a deeper understanding of pathogenesis in the metabolism and thereby provide a basis for more appropriate treatment regimens. This review describes the critical link between epigenetic modifications and TFs and their contribution to the development of obesity.

## Interaction between epigenetic modifications and TFs

Epigenetics can be described as reversible and heritable changes to the DNA that regulates chromatin structure and gene expression without altering the DNA sequence (27). The genome of each organism contains both DNA sequence information and epigenetic information. The interaction between the two specific factors maintains the function of organs and cells. Research has confirmed that there are specific genes in the body that can confer different susceptibilities to disease. However, owing to the stability of the genomic structure, most environmental factors cannot cause gene mutations or DNA sequence changes (28, 29). Therefore, some authors speculate that the changes in gene expression mediated by epigenetic modifications cause genomic abnormalities in the body during development, or result in increased susceptibility to certain diseases (30, 31). However, this process requires the participation of TFs. The currently most studied epigenetic modifications are DNA methylation, histone modification, chromatin remodeling, and non-coding RNA. The relationship between epigenetic modifications and TFs are described below.

## DNA methylation and TFs

DNA methylation is a process in which cytosine is converted to 5-methylcytosine (5-mC) by DNA methyltransferases (DNMTs) with S-adenosylmethionine (SAM) as a methyl donor (32, 33). In mammals, DNMTs comprise three active enzymes, DNMT1, DNMT3A, and DNMT3B, and one related regulatory protein, DNMT3L (33). DNMTs can promote DNA methylation and cause gene silencing. In contrast, the ten-eleven translocation (TET) enzyme is associated with DNA demethylation by reactivating the expression of the silenced gene (34). In most vertebrates, DNA methylation occurs predominantly at highly aggregated CpG dinucleotides, termed CpG islands (CGI), which overlap with gene promoter regions and can be bound by ubiquitous TFs (35). The association between methylation and TFs can be described in the following four ways.

### DNA methylation interferes with the binding of TF to target genes

**Figure 1:**
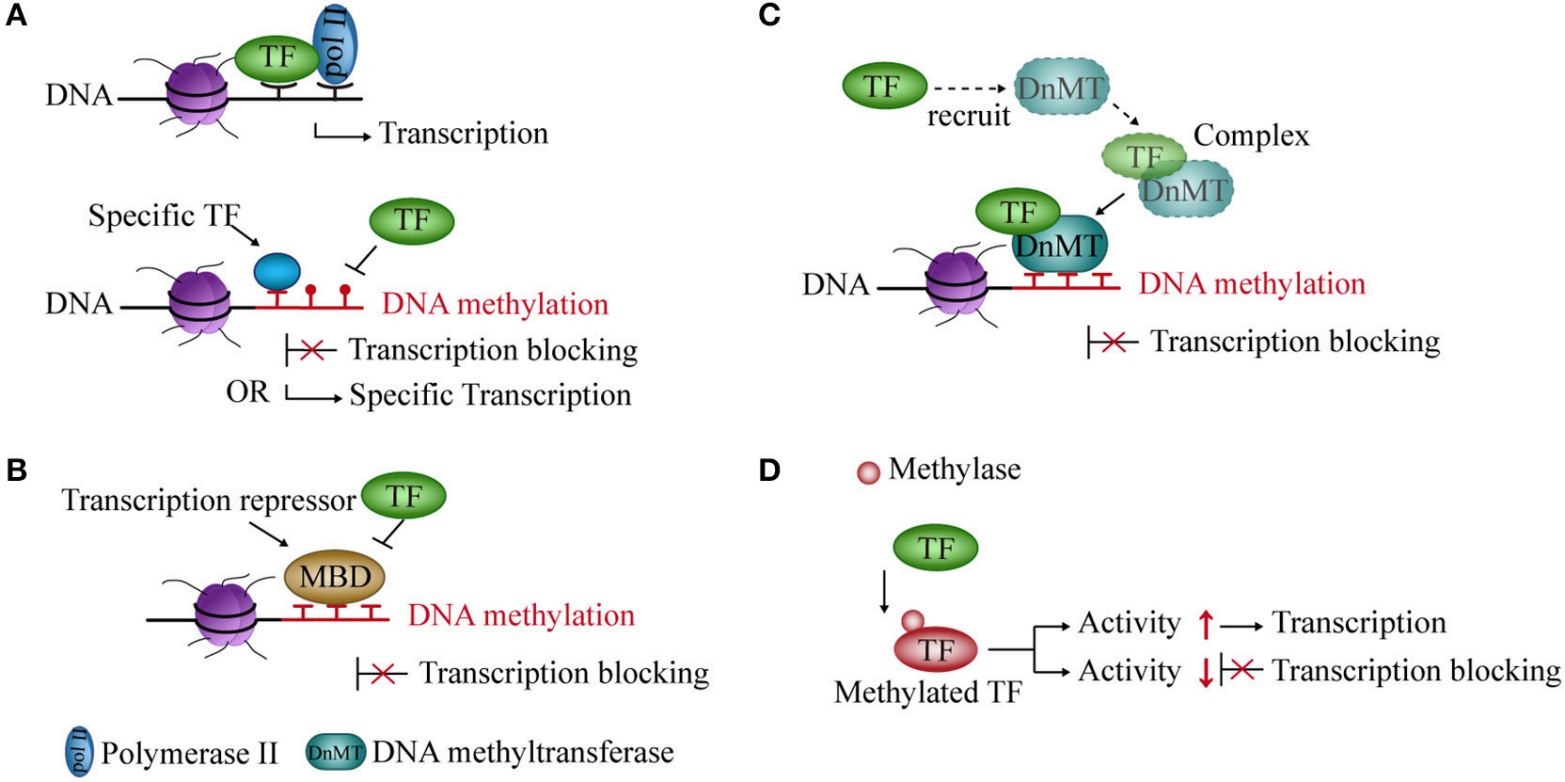
Interaction between DNA methylation and transcription factors (TFs). **(A)** Methylated DNA inhibits the binding of TFs to DNA but also creates binding sites for some specific TFs and activates transcription. **(B)** Transcriptional repressors occupy an mCpG site, thereby blocking transcription. **(C)** TFs recruit DNA methyltransferases to form a complex and repress transcription. **(D)** Methylase acts on TF and regulates its activity and transcription.

DNA methylation prevents the binding of TFs to their binding sites and influences gene transcription (36, 37). Studies showed that CpG methylation of the cAMP-responsive element (CRE) leads to loss of the binding site and of transcriptional activity of some TFs *in vitro* and *in vivo* (38). However, the methylation of the CRE sequence, which is often associated with promoters of cell type-specific genes, creates a TF binding site for C/EBPα, thereby activating transcription during adipocyte differentiation (39, 40) (Figure 1A).

### Competition between TFs and transcriptional repressors for mCpG sites

Transcriptional repressors compete with TFs for binding to methyl-CpG sites in the DNA sequence and block transcription. In mammals, one of the main transcriptional repressors are the methyl-CpG-binding domain (MBD) proteins, which comprise eleven known proteins containing the MBD domain (41, 42). These proteins are recruited to a specific sequence containing methylated cytosines, and prevent TFs and RNA polymerase from forming the initial complex at the corresponding methyl-CpG site (43, 44), thereby inhibiting gene transcription (Figure 1B).

### Association of (de)methylation enzymes and TFs in DNA methylation

The expression of DNMT and demethylase genes are affected by TFs. TFs modulate promoter methylation levels by recruiting and forming complexes with DNMTs (45). Knocking out some TFs can reduce the recruitment of DNMTs and reduce gene methylation levels in the glioma cell line (46) (Figure 1C).

### Methylation of TFs

DNA methylation can also modify TFs. Methylation of TFs belongs to non-histone methylation. Previous studies have indicated that non-histones can be methylated at lysine residues (47). For example, the RelA (p65) subunit of nuclear factor kappa-light-chain-enhancer of activated B cells (NF-κB) is methylated by lysine methyltransferase Set9 at lysine residues 314 and 315 in cells. It inhibits the activity of NF-κB and prevents it from binding to DNA, thereby causing transcriptional repression (48). In contrast, nuclear receptor-binding SET domain-containing protein 1 (NSD1, a lysine methylase) can methylate RelA at K218 and K221 and active NF-κB transcriptional activity in cell line (49) (Figure 1D).

## Histone modification and TFs

Histone modifications are another essential component of epigenetics. Histones comprise core histones (H3, H4, H2A, and H2B) and linker histones, H1/H5. Two copies of the four core histones are wrapped around ~165 bp of DNA to constitute nucleosomes and form eukaryotic chromatin with the linker histone H1 (50, 51). In histones, flexible charged tails extend from the core region as N- or C-terminal ends and can be covalently modified by various enzymes. This modification is termed post-translational modification (PTM) and may lead to changes in chromatin structure and further genome regulation. Known modifications of N-terminal tails of histones include methylation, acetylation, phosphorylation, ubiquitination, ADP ribosylation, and sumoylation (52). Histone acetylation and deacetylation are the most studied (52, 53), and are considered reversible processes controlled by histone acetyltransferases (HATs) (54) and histone deacetylases (HDAC) (55), respectively. In addition, histone methylation and demethylation are catalyzed by histone methyltransferases (HMTs) and histone demethylases (HDMs), respectively. The modification on histone tails occurs sequentially or in combination, termed “histone code” (56). This code is read by binding of specific TFs. Associations between histone modifications and TFs are divided into the following categories.

### Histone modification enzymes interact with TFs

**Figure 2:**
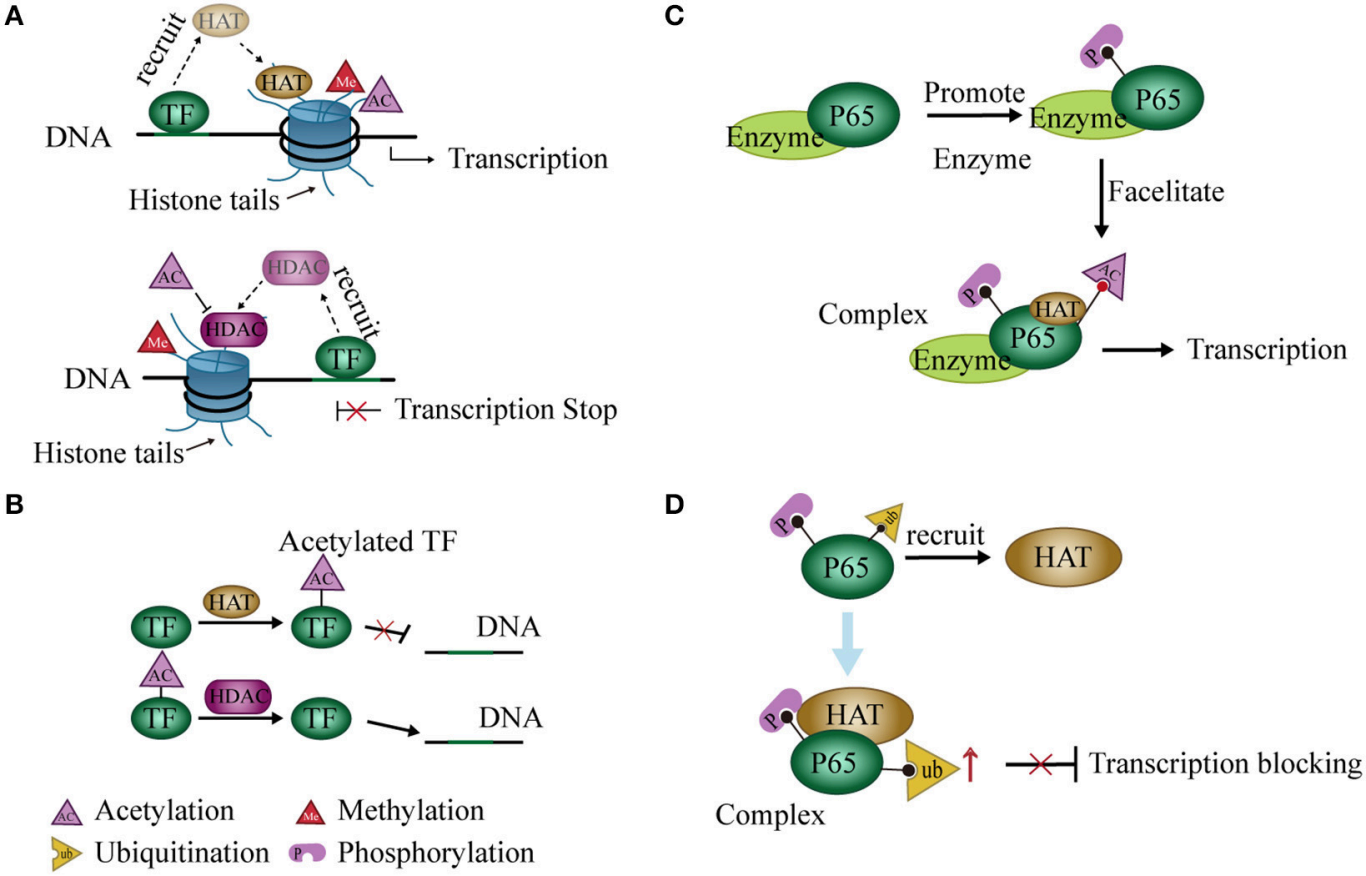
Interaction between histone modification and transcription factors (TFs). **(A)** TFs recruit histone deacetylases (HDACs) or histone acetyltransferases (HATs) and regulate gene expression by promoting histone deacetylation or acetylation, respectively. **(B)** HDACs or HATs can act on TFs and interfere with binding to DNA. **(C)** Phosphorylation at some sites of p65 by related enzymes facilitates the acetylation of other sites, thereby promoting transcription of NF-κB. **(D)** Phosphorylation of P65 recruits HATs to promote P65 ubiquitination and block transcription.

TFs act as a “porter” to recruit histone modification enzymes to regulate gene expression. For example, during histone acetylation, specific TFs repress or activate gene expression through HDAC or HAT recruitment, respectively (57). The activator or repressor role of these TFs on the promoter may depend on its binding site or time on DNA in the nuclear extracts of murine L929 cells (58) (Figure 2A). In some cases, TF recruits HATs or HDACs and then binds to these enzymes to form a complex that increases the enzymatic activity and further regulates the expression of related genes (59, 60). Changes in expression of these genes may result from changes in chromatin structure caused by histone modifications (56). The condensed state of chromatin leads to an inability of TFs to bind to target genes when histone is acetylated (61). Coincidentally, HATs may also act on TFs and induce acetylation of these proteins (62–64). Expression of related genes is regulated by the acetylation levels of these TFs (62, 65, 66) (Figure 2B).

### Crosstalk of TFs with histone modifications and PTMs

Modification of the histone code widely occurs in PTM of histone tails. A modification may either enhance or inhibit another (67–69). For instance, the phosphorylation of H3S10 leads to acetylation of H3K14 (67) but blocks acetylation of H3K9 in cell cycle progression (68). Similarly, this modification pattern may occur in TFs. For example, phosphorylation at serine residues 276 and 536 of the P65 subunit of NF-κB facilitates P65 acetylation at lysine 310 in a mammalian one-hybrid system (70, 71) (Figure 2C). On the other hand, phosphorylation of P65 also promotes P65 ubiquitination and leads to the degradation of P65 and repression of transcription in cells. However, this process may require the participation of GCN5 (a HAT) (72) (Figure 2D). Post-translational modifications of NF-κB were reviewed previously (73).

## Chromatin remodeling and TFs

Chromatin remodeling is a dynamic modification of chromatin architecture, which opens the access for regulatory transcription machinery proteins and condenses DNA to control gene expression. Remodeling is accomplished in three ways: (1) covalent histone modifications by enzymes such as HATs, HDACs, and HMTs; (2) ATP-dependent chromatin-remodeling complexes (74), such as the SWI/SNF family (BAF60a, BAF250, and BAF57), the ISWI family, the Mi-2/CHD family, and the INO80 family; and (3) utilization of histone variants (75). Histone modifications loosen or tighten the DNA wrapped around histones, and thereby allow or prevent binding of TFs to the DNA (75). In contrast, ATP-dependent chromatin-remodeling complexes have a common ATPase domain and utilize the energy of ATP hydrolysis to mobilize nucleosomes along DNA. These complexes can expel histones from DNA or facilitate the exchange of histone variants, which modulate DNA accessibility and alter nucleosome structure (76). From these mechanisms, the connection between chromatin remodeling and TFs is probably reflected in the following aspects.

### Role of pioneer factors in opening chromatin

**Figure 3:**
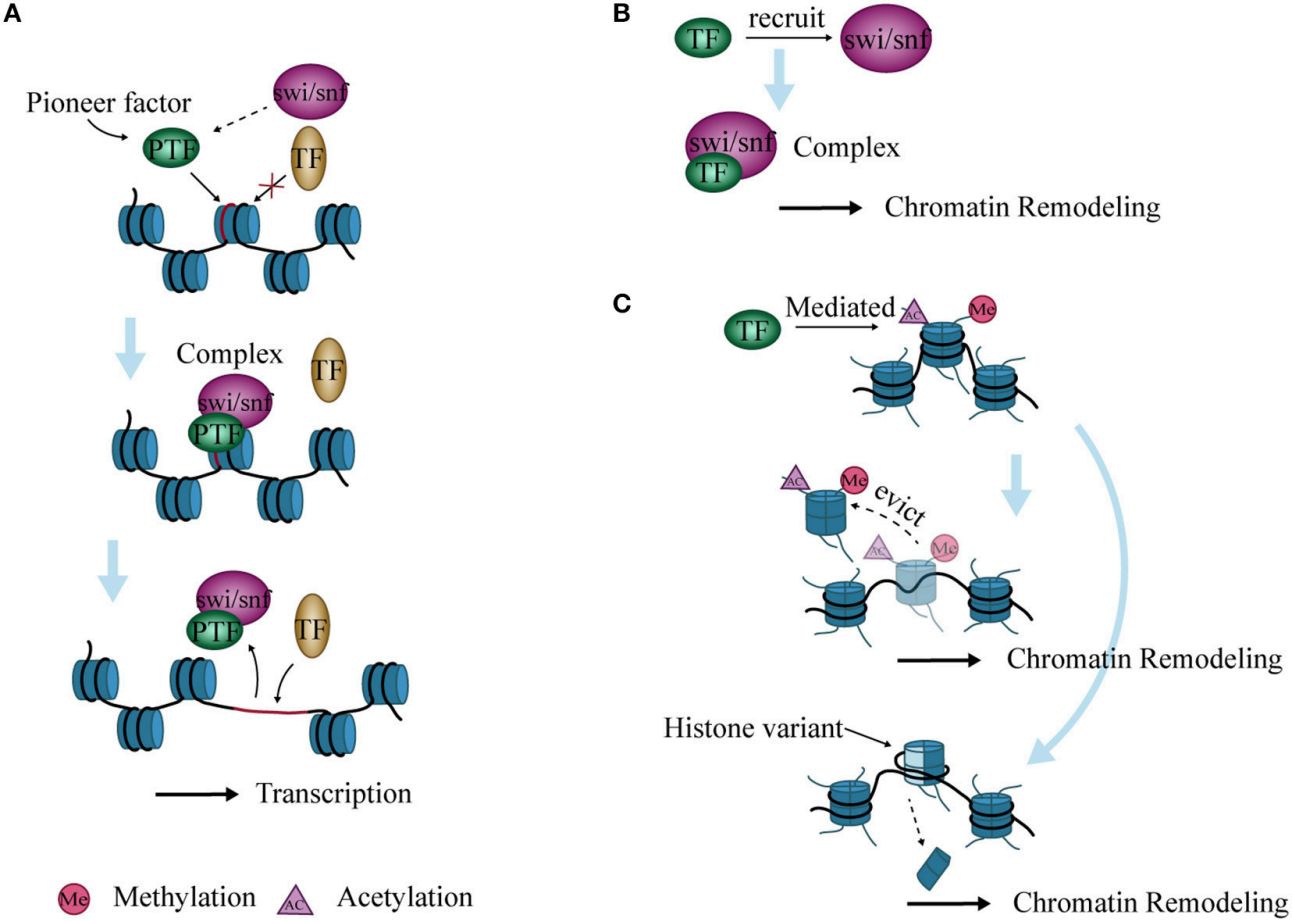
Interaction between chromatin remodeling and transcription factors (TFs). **(A)** Pioneer factors bind to specific DNA sites and form a complex with SWI/SNF to open chromatin. The complex then allows the binding of other TFs, thereby promoting transcription. **(B)** TFs recruit SWI/SNF to form complexes that lead to chromatin remodeling. **(C)** Histone variants are inserted into nucleosomes, or nucleosomes are evicted from DNA after histone modification mediated by TFs, ultimately causing chromatin remodeling.

Genes in eukaryotic cells are packaged in chromatin. TFs must reorganize local nucleosome structure and create an open site for successful interaction with genomic regulatory elements during transcription. TFs with this characteristic are termed “pioneer factors” (77). Examples include the forkhead box (FOX) proteins and the glucocorticoid receptor (GR) (78–80). These TFs recruit SWI/SNF and form a complex to open chromatin (81). The resident time of the complex is short, and the opened chromatin state is maintained for a time (82). The site then becomes accessible to other adjacent TFs to bind after dissociation of the complex (82, 83). (Figure 3A).

### Binding of TFs to chromatin remodeling complexes

TFs cannot induce chromatin remodeling independently during transcriptional regulation, but need to recruit chromatin remodeling complexes. The PRotein Interactions by Structural Matching (PRISM) algorithm shows that GR binds to several subunits of SWI/SNF such as BAF60a, BAF250, and BAF57 (84). C/EBPα and/or C/EBPβ have also been shown to recruit the SWI/SNF complex to target promoters and regulate the differentiation of adipocytes, neutrophil granulocytes, and hepatocytes in mice (85, 86) (Figure 3B).

### Histone modification by TFs induce chromatin remodeling

Chromatin remodeling is closely related to modifications of histone N-terminal tails, and these modifications affect chromatin stability (87). During histone modification, histone variants are inserted into nucleosomes that alter their structure and function, which further affect the highly ordered structure of chromatin (88). In addition, activated TFs can mediate the acetylation of histones and evict nucleosomes, which remodel chromatin and induce transcription initiation or elongation *in vitro* (89) (Figure 3C).

## Non-coding RNA and TFs

It is well known that < 2% of transcripts in the mammalian genome have a protein-coding function, with the remaining 98% being non-coding RNA (ncRNA) (90). ncRNA are mainly classified into two types: microRNA (miRNA) and long non-coding RNA (lncRNA). Studies have found that the promoter region of miRNA and lncRNA genes can contain different epigenetic modifications and are involved in many biological processes (91–94) through the interaction with TFs. Any abnormality in these transcriptional processes can lead to disease (95).

### Interaction between miRNA and TFs

**Figure 4:**
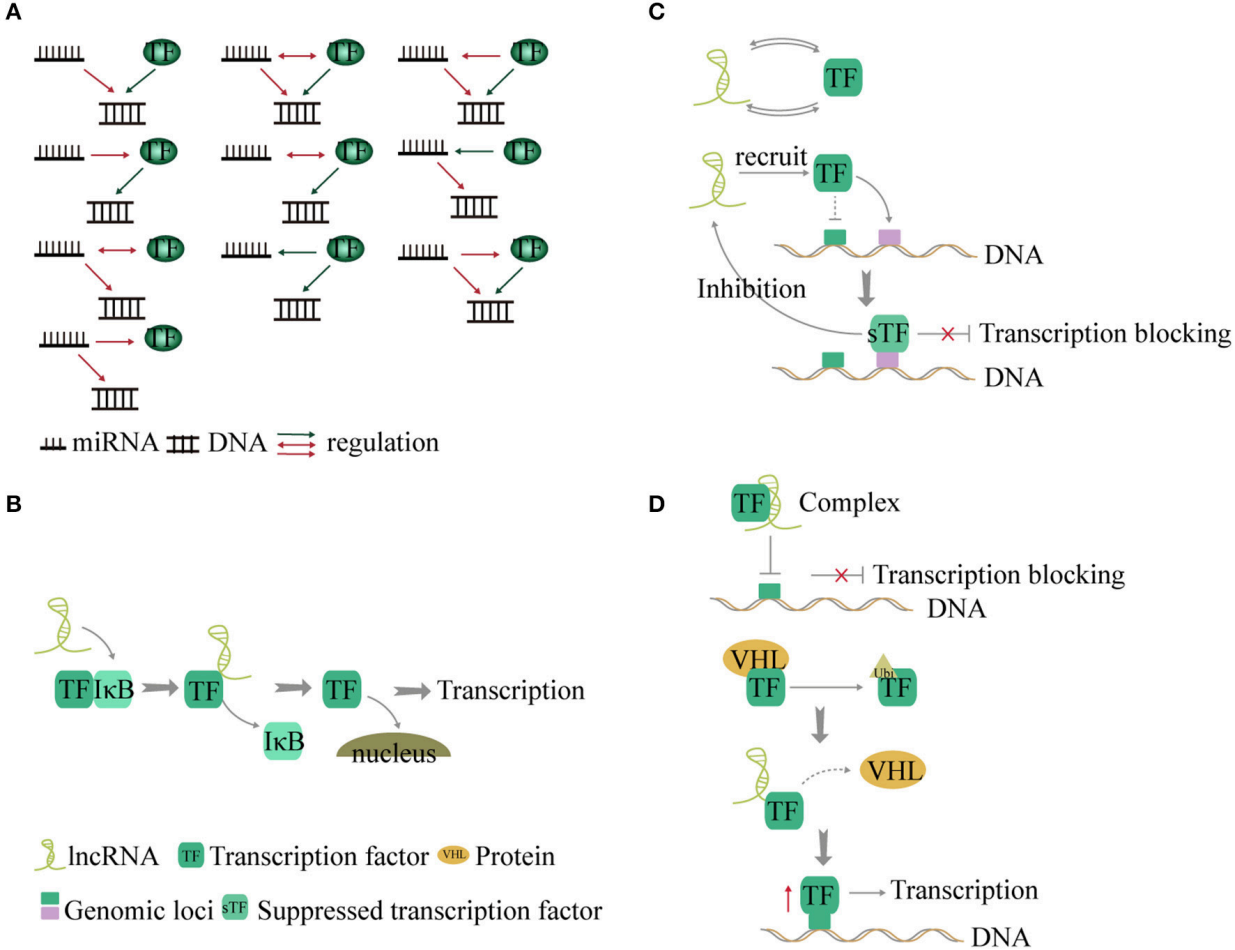
Interaction between non-coding RNA and transcription factors (TFs). **(A)** TF and miRNA present a feed-forward loop pattern. There are 10 modes of interaction between TF, miRNA, and genes. **(B)** LncRNAs act as co-factors or inhibitors to control some transcription factor-related signaling pathways. **(C)** LncRNAs and TFs interact with each other to regulate transcription by forming a binary network. In addition, lncRNA recruits TF to other gene loci and blocks transcription. Conversely the suppressed TF inhibits the expression of the lncRNA. **(D)**. LncRNA forms a complex with TF to suppress gene transcription. Additionally, lncRNA disrupts the interaction between TF and the VHL protein and forms a complex with TF to reduce the levels of TF ubiquitination, thus increasing the expression of TF and promoting gene transcription.

TFs and miRNA play an important role in gene transcription and post-transcriptional regulation. Studies found that genes regulated by the same TF and miRNA present a significant co-expressed pattern (96, 97). During regulation, TFs bind to the upstream promoter region of the miRNA gene and exert an influence on transcription. In addition, some miRNAs conversely affect translation of TFs (98). Therefore, TFs and miRNA make up a complex regulatory network termed feed-forward loop (96) in stabilizing gene regulation (9). Ten kinds of interactions have been described between TFs, miRNA, and genes (9, 99) (Figure 4A).

### Interaction between lncRNA and TFs

LncRNAs can alter the transcription of genes by interacting with TFs, chromatin-modifying complexes, or mRNA (100, 101). The interaction between lncRNA and TFs can be divided into the following categories.

- LncRNAs act as co-factors or inhibitors to regulate the activity of TFs and control NF-κB signaling (102). LncRNA-Cox2 promotes degradation of IkBa in the cytoplasm (102). Knock out of lncRNA-Cox2 leads to reduced expression of NF-κB (102). However, TFs can also affect lncRNA expression (103). The roles of lncRNAs in cancer metabolism were identified as energy stress-induced lncRNA by FoxO transcription factors (104) (Figure 4B).
- TFs and lncRNAs form a binary network and combine with genes to form a ternary complex (105, 106). In a recent study, 530 TF-lncRNA pairs were identified in the cell cycle (107). In a trans-acting network, lncRNAs regulate TF-mediated chromatin remodeling and transcription (108). In addition, lncRNA can recruit one TF to the genomic loci of another TF and inhibit gene transcription (109). The suppressed TF, in turn, inhibits the expression of the lncRNA (109). The TF-lncRNA-gene-network shows that 69 genes and lncRNA are controlled by TFs (100) (Figure 4C).
- LncRNA can form complexes with TFs to regulate transcription of target genes (110, 111). For example, Lethe, a lncRNA, binds to RelA and prevents it from binding to the NF-κB target promoter, thereby inhibiting transcription of proinflammatory cytokine genes (112). However, binding of lncRNA-p21 to HIF-1a, a key TF that mediates the response to hypoxia, can disrupt the interaction with the Von Hippel-Lindau (VHL) protein and cause high levels of HIF-1a expression (113). This is a positive feedback loop between TF and lncRNA (Figure 4D).

## Role of TFs in epigenetic regulation in the development of obesity

### Role of PPARγ in obesity

PPARγ is a critical transcriptional regulator of adipogenesis in mammals, is closely related to regulation of lipids and glucose metabolism, and is associated with the control of obesity and related diseases (114, 115). Siersbaek et al. analyzed the PPARγ and C/EBPα binding sites and found that PPARγ and C/EBPα regulate the expression of most genes associated with adipogenesis (116). The BPro12Ala and 6CAC478CAT exon polymorphisms of the PPARγ gene are significantly related to the incidence of severe obesity (117). In the USA, mutations in the PPARγ gene can severely cause certain types of sexual obesity in women (118). Animal studies also provide more direct evidence for research in this area. For example, Japanese researchers reported that, in high-fat diets, mice lacking PPARγ exhibited significant dysplasia of adipocytes, smaller cell morphology, and lower fat content, causing pathological features such as obesity and severe insulin resistance (119). PPARγ not only promotes the proliferation and differentiation of adipocytes but also confers insulin sensitivity to adipocytes (120). An increase in insulin sensitivity can in turn promote the expression of the PPARγ gene in adipose tissue, thereby positively accelerating the differentiation of adipocytes. Both these aspects greatly increase the efficiency of adipocyte synthesis and storage and are important factors for obesity.

In addition, PPARs also play an important role in lipid metabolism. After feeding, carbohydrates and fats are converted into glucose and chylomicrons, respectively, and enter circulation. Most of the glucose is absorbed by the liver. If hepatic glucose storage capacity is reached, the remaining glucose is used for lipogenesis. Studies have shown that rats in the fed state increase the amount of SREBP1, which promotes the conversion of glucose to acetyl-CoA, followed by synthesis of fatty acids. Fatty acids are further converted into triglycerides and very low-density lipoproteins and are stored in the body (120, 121). This may result from the fact that PPARγ is a target gene for SREBP1 (122), which is involved in the production of endogenous PPARγ ligands (possibly fatty acids). SREBP1 stimulates the uptake of glucose and fatty acids, which are subsequently converted to triglycerides (14, 15).

## Role of PPARγ in epigenetic factors in adipose tissue

### DNA methylation and PPARγ

Studies have shown that DNA methylation regulates the expression of PPARγ. Two studies examined the CpG methylation of the PPARγ gene in 9–year–old overweight children (123, 124), and both confirmed the presence of different levels of methylation. In addition, one group pointed out that the level of CpG methylation is negatively correlated with the expression of PPARγ (124), whereas the other group suggested that PPARγ methylation levels may be related to child body size, with an upward trend with age (123).

Studies on PPARγ in animals have revealed that the PPARγ2 gene promoter is highly methylated in mouse 3T3-L1 pre-adipocytes (125). However, with the differentiation of 3T3-L1 pre-adipocytes, the PPARγ2 promoter is gradually demethylated, and the expression of PPARγ2 mRNA is gradually increased. The DNA methylation inhibitor 5-aza-2′-deoxycytidine interferes with the normal differentiation of 3T3-L1 pre-adipocytes and inhibits lipid accumulation in adipocytes (125). Compared with wild-type mice, methylation levels of the PPARγ2 promoter in visceral adipocytes increase, whereas PPARγ2 mRNA levels decrease in obese diabetic mice (125). This suggests that DNA methylation of the PPARγ promoter can inhibit the expression of the PPARγ gene and is associated with the occurrence of obesity and diabetes. However, no studies mentioning the involvement of DNMT or demethylase in the regulation of PPAR gene during fat differentiation or obesity were found.

### Histone modification and PPARγ

Histone methylation regulates PPARγ gene expression. Several HMTs and HDMs are known to be involved in the regulation of PPARγ expression and lipogenesis (126). Myeloid/lymphoid or mixed-lineage leukemia protein 3 (MLL3) and myeloid/lymphoid or mixed-lineage leukemia protein 4 (MLL4) are major H3K4 methyltransferases in mammalian cells (126). Studies have shown that MLL4 can bind to the PPARγ locus and increase PPARγ gene expression and lipogenesis. The early adipogenic TF C/EBPβ serves as a pioneer TF and recruits MLL4 to the PPARγ gene loci and induces PPARγ and C/EBPα expression. MLL4 is then recruited to cooperate with other adipogenic TFs for adipocyte gene expression (126). In 3T3-L1 pre-adipocytes and mature adipocytes, the TF tonicity-responsive enhancer binding protein (TonEBP) binds to the PPARγ2 promoter directly, giving rise to H3K9me2 in the PPARγ2 promoter, thereby inhibiting PPARγ2 activity (127). PPARγ2 expression decreases, thereby inhibiting adipogenesis. The binding of TonEBP to the PPARγ2 promoter is associated with blocking C/EBPβ binding to H3K9m2 (127). In addition, PPARγ is also regulated by SET domain family proteins (128). Importantly, SET domain bifurcated 8 (SETD8) is a direct target of PPARγ and is induced during adipocyte differentiation. SETD8 regulates many target genes that depend on PPARγ activation by increasing the monomethylation levels of H4K20 during adipogenesis (128).

Histone acetylation also regulates PPARγ expression. It was found that the epigenetic modification of the PPARγ gene is regulated by C/EBPs and GR *in vitro* in a lipogenic cell model (129). The transient recruitment of GR and C/EBPβ by a complex consisting of MED1 and p300 (HAT) in the enhancer region of PPARγ2 causes a significant increase in the levels of H3K9 acetylation in this region, which enhances the expression of PPARγ2 and becomes a major driver of adipogenesis (130). Analysis at the genome level revealed that, during differentiation of 3T3-L1 cells, H3K9 and H3K27 acetylation at the PPARγ locus increases significantly, and both acetylation at both sites are positively correlated with the expression of the PPARγ gene. However, the roles of H3K9 and H3K27 acetylases in lipogenesis and PPARγ expression have not been determined (131). In contrast to the functions of HATs, HDACs repress PPARγ expression by deacetylating histones (132). Several HDACs are significantly downregulated during adipogenesis (133). SIRT1, a class III NAD-dependent HDAC, is an inhibitor of PPARγ (134). *In vivo*, fasting-induced SIRT1 binds to the PPARγ binding site of a fat-specific gene, enhancing its inhibitory function and thereby blocking adipogenesis (134). Studies have also confirmed that SIRT1 plays a role in the browning of white adipose tissue (135). This may be related to the deacetylation effect of SIRT1 on the regulation of PPARγ, whereas PPARγ promotes the production of brown fat by inducing the transcription-assisted regulator PRDM16 (135).

### Chromatin remodeling and PPARγ

During lipogenesis, the chromatin environment at the PPARγ locus also needs to be correctly remodeled to allow expression of PPARγ target genes. Within a few hours after differentiation of 3T3-L1 preadipocytes, chromatin remodeling occurs in the PPARγ locus leading to an open state (129). This chromatin opening may be related to histone modifications at the PPAR locus and binding of pioneer TFs (127). Studies have shown that remodeling and opening of the PPARγ2 promoter region are dependent on protein kinase A (PKA). Knockout of the PKA gene using shRNA results in decreased chromatin accessibility in the PPARγ2 promoter region (136). In addition, studies have shown that specific binding to and function of the PPARγ promoter in adipocytes requires the participation of G-protein suppressor 2 (GPS2) (137). GPS2 primes a local chromatin environment via inhibition of the ubiquitin ligase RNF8 and stabilization of the H3K9 histone demethylase KDM4A/JMJD2 (137). Moreover, the SWI/SNF chromatin remodeling complex regulates PPARγ2 expression during adipogenesis. PPARγ activity also depends on components of a chromatin remodeling SWI/SNF complex with ATPase BRG1 and BAF60c subunits (138). A dominant mutant of Brg1 inhibits the transdifferentiation of fibroblasts into adipocytes induced by PPARγ, C/EBPα, and C/EBPβ (139). However, it remains unclear how Brg1 is recruited to the PPARγ promoter.

Chromatin remodeling can be regulated by replacement of canonical histones by histone variants. Genome-wide localization studies have shown that the variant H2A.Z, which is mainly involved in the regulation of gene expression, can be preferentially located in the promoter and enhancer regions (140). Studies have shown that, during adipogenesis, the E1A-binding protein p400 complexed with subunit bromo-containing protein 8, the p400/Brd8 complex, influences the expression of PPARγ target genes by inserting the histone variant H2A.Z into the transcriptional regulatory region (141). This is crucial for differentiation of fat tissue.

### Non-coding RNA and PPARγ

Non-coding RNA is an important post-transcriptional gene expression regulator that modulates many physiological and pathological processes (142). Multiple obesity-associated miRNAs have been identified in adipose tissue in obese humans, rats, and mice (143, 144). Several of these miRNAs can regulate PPARγ, and are classified into two categories: inhibition and stimulation of PPAR transcription (Table 1). Five miRNA can bind to the 3′ UTR of the PPARγ gene mRNA or interact with PPARγ at the cellular level to inhibit PPARγ protein expression and adipocyte differentiation (142, 145–151). In contrast, miR-103, miR-143, miR-200a, miR-335, and miR-375 have a lipogenic effect during adipocyte development resulting from elevated PPARγ expression (152–156). The ectopic expression of miR-103 increases the accumulation of triglycerides in adipocytes and up-regulates the expression of PPARγ2 (152). MiRNA, PPARγ gene, PPARγ, and related transcription factors form a cyclic network that regulates fat tissue formation.

**Table 1:** Regulation of PPARγ by miRNAs during adipogenesis and obesity.

**miRNA**	**Function**	**Species**	**Experimental system**	**References**
miR-27a miR-27b	↓adipogenesis	Obese mice	3T3-L1 pre-adipocytes	(145, 146)
		Human	Human multipotent adipose-derived stem cells.	(147)
miR-130 miR-130b	↓adipogenesis	Human	3T3-L1 pre-adipocytes	(148)
		Obese mice	Epididymal adipose tissue	(149)
miR-301a	↓adipogenesis	Obese mice	3T3-L1 pre-adipocytes	(142)
miR-302a	↓adipogenesis	Obese mice	3T3-L1 pre-adipocytes	(150)
miR-548d-5p	↓adipogenesis	Human	Human bone marrow mesenchymal stem cells	(151)
miR-103	↑adipogenesis	Obese mice	3T3-L1 pre-adipocytes	(152)
miR-143	↑adipogenesis	Obese mice	3T3-L1 pre-adipocytes	(153, 152)
miR-200a	↑adipogenesis	Yak	Separate cells from perirenal adipose tissue	(154)
miR-335	↑adipogenesis	Obese mice	3T3-L1 adipocytes	(155)
miR-375	↑adipogenesis	Obese mice	3T3-L1 pre-adipocytes	(156)

*↑ Promotes lipogenesism, ↓ Inhibits lipogenesis*.

In the process of 3T3-L1 cell differentiation, lncRNA can also affect lipogenesis by affecting PPARγ transcription. It was found that, in 3T3-L1 adipocytes, lncRNA U90926 inhibits the activity of the PPARγ2 promoter, thereby inhibiting PPARγ expression and lipogenesis (157). In contrast, lncRNA NEAT1 plays a regulatory role in alternative splicing events of PPARγ during differentiation of mouse 3T3-L1 cells. Interference with NEAT1 expression up-regulates PPARγ2 expression and promote fat tissue differentiation (158). In addition, overexpression of lncRNA HOTAIR promotes the expression of PPARγ and the differentiation of pre-adipocytes into mature adipocytes, although the mechanism remains unclear (159). The mechanism underlying the role of lncRNA in lipogenesis and obesity still needs to be further investigated.

## Participation of PPARs in epigenetic modulation of lipid metabolism

Fatty acids can be oxidized to CO_2_ and H_2_O in the presence of oxygen, releasing large amounts of energy and becoming one of the main energy sources for the body. Most of the fatty acids in the human body are exogenous fatty acids derived from foods and can be utilized through transformation and processing. The body can also convert sugars and proteins into endogenous fatty acids and triglycerides to store energy. Fatty acid synthase catalyzes the first step in de novo lipogenesis.

PPARs are ligand-activated receptors that heterodimerize with the retinoid X receptor (RXR), bind reactive elements in target genes, and induce transcription. PPARγ can modulate fatty acid metabolism, promote differentiation of adipocytes, and regulate lipid storage through transcription of a series of lipid-related proteins in the posterior segment (160). In animals, it has been demonstrated that fatty acids act as ligands for PPARs and activate the expression of genes involved in fatty acid metabolisms such as the transmembrane proteins CD36, lipoprotein lipase, fatty acid-binding protein 4, and long-chain acyl-CoA synthetase 1 (161). Studies have found that multiple miRNAs are involved in the regulation of fatty acids. MiR-122 is the first miRNA confirmed to be involved in lipid regulation and is the most abundant miRNA identified in liver tissue (162). MiR-122 plays a prominent role in maintaining the phenotype of liver cells and fatty acid metabolism (162). In mice, inhibition of miR-122 expression can lead to a sustained reduction in plasma cholesterol levels, increased hepatic fatty acid oxidation, and decreased liver fatty acid and cholesterol synthesis rates. Ultimately, high-density lipoprotein and apolipoprotein A1 levels increase, and low-density lipoprotein and apolipoprotein B levels are reduced (163). In addition, inhibition of miR-205-5p expression in genetically improved farmed tilapia liver can increase fatty acid synthase and PPARα mRNA levels and thus regulate hepatic lipid metabolism (164). Increased PPARα levels have been shown to increase liver fatty acid oxidation and reduce circulating triglyceride levels to regulate rodent obesity (165). In addition to miRNA regulation, studies in rodents have shown that decreased PPARα promoter methylation contributes to enhanced expression of carnitine palmitoyl transferase-1, a regulatory enzyme in fatty acid oxidation, and decreased expression of fatty acid synthase (166). However, no PPAR-mediated lipid metabolism modulation by mechanisms such as histone modification and chromatin remodeling has been described. Another important transcription factor, SREBP1, also plays an important role in lipid metabolism. NAD-dependent deacetylase sirtuin-1(SIRT1) can remove acetyl groups from lysine at positions 289 and 309 of SREBP-1c, inhibiting SREBP-1c activity, decreasing its stability, and reducing lipid production (167).

## Possible therapeutic direction in the future

**Figure 5:**
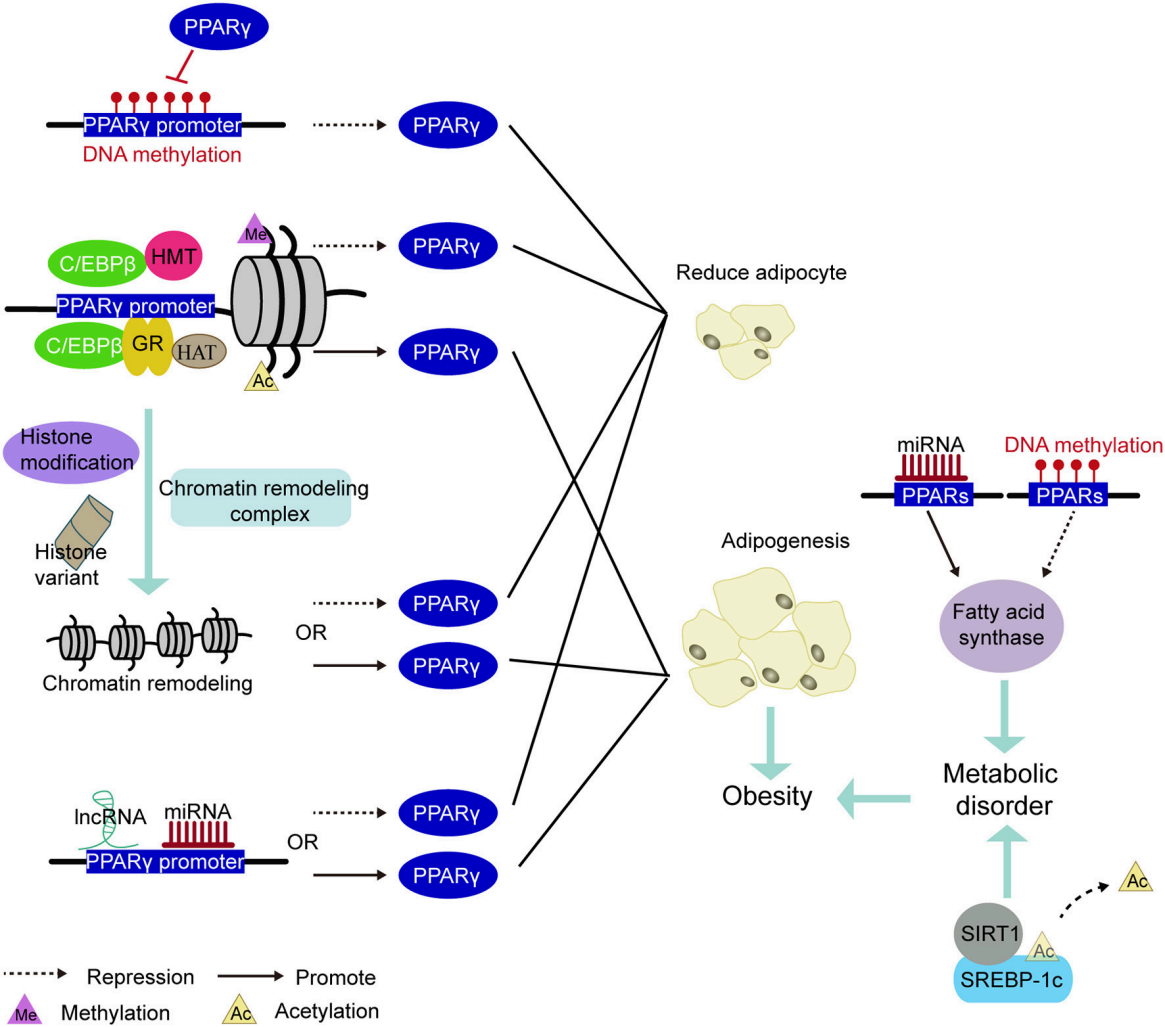
PPARs be involved in the mechanism of epigenetic modification leading to obesity. Methylation of the PPARγ promoter region inhibited PPARγ expression to decrease adipogenesis. The pioneering transcription factor (e.g., C/EBPβ or GR) recruit histone acetyltransferases or histone methyltransferases in the vicinity of the PPARγ promoter and cause histone methylation or acetylation, thereby regulating PPARγ expression. Under various factors such as histone modifications, histone variants, and chromatin remodeling complexes, the chromatin remodeling in the PPARγ promoter induces changes in the expression of PPARγ. The miRNA and lncRNA acting on the PPARγ promoter region also govern the expression of PPARγ. Inhibited expression of PPARγ reduces the production of adiposity. Conversely, promoting the expression of PPARγ increases in adipogenesis and leads to obesity. In addition, methylation and miRNA modification of the PPARs promoter region regulates the expression of fatty acid synthase and participate in lipid metabolism. Deacetylation of the transcription factor SREBP-1c is some other important way in regulating lipid metabolism. Disorders of lipid metabolism can also induce obesity.

PPAR is a pivotal regulator of fat formation and metabolism. Knocking out PPARγ in adipose tissue of mice can prevent high-fat diet-induced obesity and insulin resistance (168). Because of its complex and diverse biological functions, it has grown up to be a therapeutic target for obesity-related diseases (114). For example, PPARγ agonists have been applied to treat hyperlipidemia, hyperglycemia, diabetes (114, 169). However, these drugs, such as Thiazolidinedione (TZD), have reduced insulin resistance but also increased lipid accumulation in skeletal muscle by promoting adipocyte hypertrophy and hyperplasia (170). Therefore, long-term use of PPARγ agonists may trigger unexpected effects on systemic metabolism, including insulin sensitivity. Other more appropriate treatments are needed. With the development of epigenetics, epigenetic markers have become a useful tool to assess the risk of obesity and metabolic disorders (171). Perhaps, in the future, the epigenetic modification of PPARγ may be intervened to regulate the expression of genes involved in lipogenesis without compromising metabolism *in vivo*, thereby reducing the occurrence of obesity and obesity-related diseases (Figure 5).

## Conclusion

A variety of physiological, biochemical, genetic, and behavioral factors can cause obesity. To control the occurrence of this disease, it is necessary to reasonably control the diet and perform a moderate amount of exercise so as to reduce the storage of residual energy within the body. In addition, it is also necessary to control the excessive proliferation and differentiation of adipocytes. Knowledge of epigenetic modifications provides novel insights into the pathogenesis of obesity. Owing to the importance of PPARγ in lipogenesis and lipid metabolism in humans and other animals, the epigenetic mechanisms underlying transcriptional regulation of PPARγ will remain a research focus in the field of lipid biology and medicine and may result in improved treatments for obesity. Although much evidence for epigenetic regulation has been reported in recent years, few studies have shed light on the role of epigenetic modifications, transcription factors, and upstream and downstream mechanisms involved in obesity. In the future, a better understanding of the interrelationship between regulation of obesity-related transcription factors and epigenetics will be needed to explore effective therapeutic methods for the treatment of obesity using epigenetic modifications.

## Author contributions

QH and CM prepared the first draft and wrote the final version of the manuscript. LC and DL were involved in literature searches. FL and RC critically revised the manuscript and gave constructive opinions on articles.

### Conflict of interest statement

The authors declare that the research was conducted in the absence of any commercial or financial relationships that could be construed as a potential conflict of interest.
